# Sustainable Biochars from Agri-Food Residues for the Selective Removal of Pharmaceuticals: Performance Under Untreated Wastewater Conditions

**DOI:** 10.3390/molecules31162867

**Published:** 2026-08-17

**Authors:** Antón Puga, M. Ángeles Sanromán, Cristina Delerue-Matos, Marta Pazos

**Affiliations:** 1 REQUIMTE/LAQV, Instituto Superior de Engenharia do Porto, Instituto Politécnico do Porto, Rua Dr. António Bernardino de Almeida, 431, 4249-015 Porto, Portugal; cmm@isep.ipp.pt; 2 BIOSUV Group, Department of Chemical Engineering, CINTECX, University of Vigo, Campus Lagoas-Marcosende, 36310 Vigo, Spain; sanroman@uvigo.es (M.Á.S.); mcurras@uvigo.es (M.P.)

**Keywords:** agri-food waste-derived biochar, adsorption, pharmaceutical compounds, real wastewater matrix

## Abstract

In this study, biochars derived from agri-food wastes, including different avocado residues (peel- and seed-derived fractions), olive pits and cherry stones, were evaluated as low-cost and sustainable adsorbents for the removal of the emerging contaminants sulfamethoxazole (SMX), antipyrine (ANT) and trazodone (TRZ) from aqueous solutions. Despite their very low BET surface areas, the biochars exhibited notable adsorption capacities, indicating that adsorption performance was mainly governed by surface chemistry rather than textural properties. FTIR analysis confirmed the presence of abundant oxygen-containing functional groups on the biochar surfaces, enabling electrostatic interactions, hydrogen bonding and dipole–dipole interactions with the target contaminants. Adsorption kinetics for all systems were well described by the pseudo-first-order model, with equilibrium capacities closely matching experimental values, suggesting rapid and reversible surface interactions. Intraparticle diffusion analysis revealed that diffusion was not the sole rate-limiting step, with external mass transfer and surface interactions dominating the initial adsorption stage. Equilibrium isotherm analysis showed that SMX and ANT adsorption onto avocado-derived biochars followed the Langmuir model, whereas TRZ adsorption was better described by the Freundlich model. In contrast, olive pit biochar exhibited a markedly higher affinity for TRZ, achieving near-complete removal and fitting the Langmuir isotherm. Finally, high removal efficiencies were maintained when applying the selected biochar to real municipal wastewater, demonstrating robustness under complex matrix conditions. Overall, the results highlight the potential of these agri-food waste-derived biochars, including multiple avocado fractions, as sustainable and economically viable adsorbents for quaternary wastewater treatment.

## 1. Introduction

Solutions for wastewater treatment should include the implementation of simple and effective technologies capable of meeting increasingly stringent regulations [1,2], such as those imposed by European water legislation [3], which requires efficient treatments for the removal of emerging contaminants from water [4,5]. This need arises because conventional wastewater treatment plants (WWTPs) are not sufficiently effective for certain classes of pollutants, particularly emerging contaminants, which comprise diverse families, with pharmaceuticals being among the most prominent [6,7]. As a result, these compounds are discharged into receiving water bodies and are being increasingly detected in water quality monitoring studies [8].

To address this environmental challenge and comply with regulatory requirements, adsorption has emerged as a rapid and effective technology for wastewater purification [9,10]. Although adsorption has been known and applied for decades, it has recently regained significant attention due to its potential for large-scale implementation [11,12]. Importantly, this technology can be framed within the principles of the circular economy, offering not only an environmentally sound treatment option but also the potential to generate economic value and commercial opportunities [13,14,15].

It is well recognized that societal challenges are more effectively addressed when economic viability accompanies technical solutions. In this context, adsorption using recycled materials, specifically the reuse of waste streams, represents a key strategy to enhance the technological, economic, and environmental sustainability of water treatment processes [16].

Recent studies have reported high removal efficiencies using waste-derived adsorbents that are subjected to suitable pre-treatments prior to application [17]. Among these materials, biochar has attracted particular interest due to its favourable adsorption properties, including its porosity and capacity to retain a wide range of contaminants [18].

Based on this background, the present work explores the potential use of waste materials derived from two fruits [19], avocado and green olive. In addition to avocado seeds, several other avocado fractions were investigated, including the layer covering the seed and the outer peel of the fruit. These different components were carefully separated, pyrolyzed to produce biochars, and subsequently evaluated for the adsorption of three pharmaceutical emerging contaminants: sulfamethoxazole (a widely used antibiotic [20]), trazodone (an antidepressant that has been increasingly used since the COVID-19 pandemic [8]), and antipyrine (an analgesic/anti-inflammatory commonly employed for ear-related conditions, mainly in the form of ear drops [21]). The adsorption performance was assessed under controlled laboratory conditions and further validated by using real municipal WWTP, to evaluate the robustness and practical applicability of the developed biochars in complex matrices [22]. The adsorption processes were comprehensively analysed through kinetic and equilibrium modelling, together with an in-depth characterization of the materials.

## 2. Materials and Methods

### 2.1. Reagents

Sulfamethoxazole (SMX, 99% purity), trazodone (TRZ, 99% purity), and antipyrine (ANT, 97.5% purity), used as model pollutants and whose characteristics are presented in Table 1, were purchased from Sigma-Aldrich (St. Louis, MO, USA). Acetonitrile (ACN) was purchased from VWR Chemicals BDH Prolabo (Leuven, Belgium). Methanol (>99.9% CH_3_OH) and ammonium formate (NH_4_HCO_2_, 99%), used for the preparation of the HPLC mobile phases, were supplied by ACROS Organics (Geel, Belgium) and Fisher Scientific (Loughborough, UK), respectively. All reagents were of analytical grade. Milli-Q water was used for the preparation of all solutions, except for the experiments performed with wastewater collected from the influent of a WWTP located in southern Galicia, Spain (see Section 2.6 for further details).

### 2.2. Biochar Preparation

**Figure 1:**
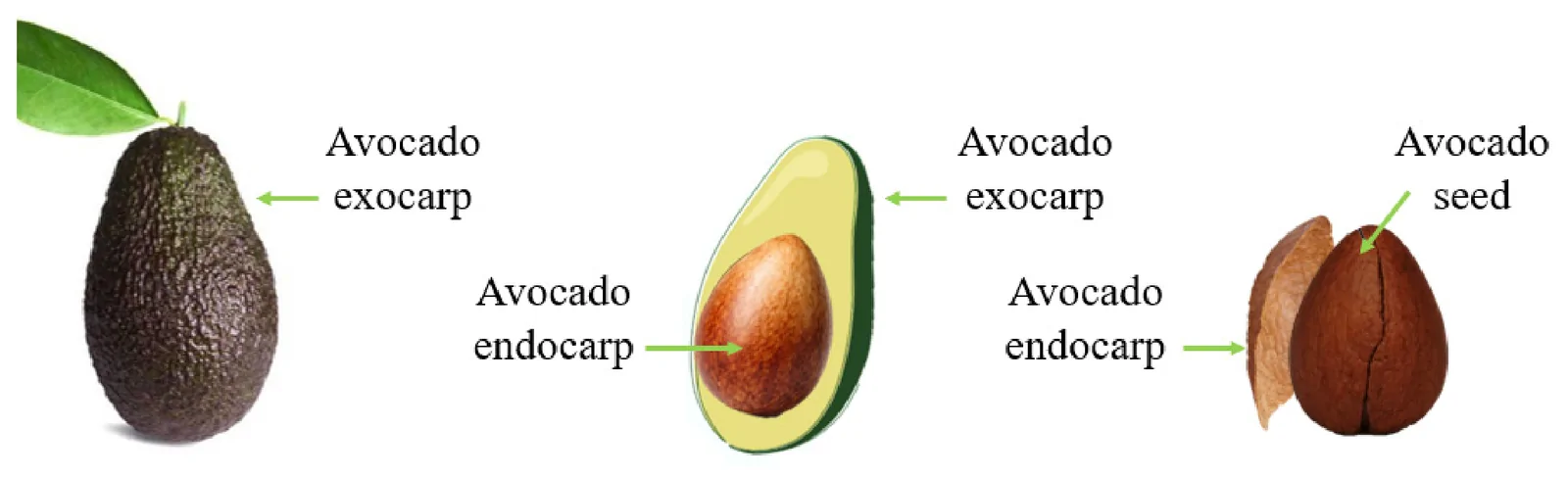
Different parts of the avocado used as waste material for biochar production, namely: avocado exocarp (AEx), endocarp (AEn) and, seed (AS).

Different avocado residues (Figure 1)—exocarp (AEx), endocarp (AEn), seed (AS) and scraped seed (SAS); olive pits (OP) and cherry stones (CS)—were dried in an oven at 65 °C for 48 h. Then, biochar was produced by thermal decomposition of the raw waste material at 400 °C for 2 h in a muffle furnace under an inert nitrogen atmosphere, applying a heating rate of 10 °C min^−1^. In the case of the SAS, it was obtained from AS by manually removing the outermost layer, approximately 1–2 mm thick.

### 2.3. Characterization

A comprehensive physicochemical characterization of the biochars was conducted. Surface morphology was examined by Scanning Electron Microscopy (SEM) using a JEOL JSM-6700F (JEOL Ltd., Akishima, Tokyo, Japan) microscope equipped with an Oxford Inca Energy 300 EDS system (Oxford Instruments, Abingdon, Oxfordshire, UK), operated at an accelerating voltage of 15 kV (Electron Microscopy Service, C.A.C.T.I., University of Vigo). The specific surface area was calculated according to the Brunauer–Emmett–Teller (BET) method from N_2_ adsorption data obtained with a Micromeritics ASAP 2020 instrument (Micromeritics Instrument Corporation, Norcross, GA, USA) (C.A.C.T.I., University of Vigo). Fourier-transform infrared (FTIR) spectra were recorded on a Jasco FT/IR-4100 spectrometer (JASCO Corporation, Hachioji, Tokyo, Japan). Prior to analysis, the samples were dried at 60 °C for 40 min (C.A.C.T.I., University of Vigo). The point of zero charge (pH_PZC_) was determined by the mass titration method [12].

### 2.4. Quantification of Pharmaceuticals

The concentration of the pharmaceuticals, either individually or in mixture, was determined using an HPLC system equipped with a diode array detector (DAD) (Agilent 1260 Infinity: Agilent Technologies, Santa Clara, CA, USA) and a Kinetex LC column (5 µm Biphenyl, 100 Å, 150 × 4.6 mm) (Phenomenex, Torrance, CA, USA). The mobile phase consisted of ultrapure water (65%, *v*/*v*), methanol (30%, *v*/*v*), and ammonium formate solution (100 mM) (5%, *v*/*v*), with a flow rate of 1 mL/min. The column was kept at room temperature, and the maximum detection wavelengths were: SMX (266 nm), ANT (242 nm) and TRZ (246 nm). More detailed analytical procedures can be found in Hayoun et al. [23].

### 2.5. Adsorption Test

#### 2.5.1. Preliminary Experiments

The preliminary biochar adsorption studies were performed in 200 mL flasks, with 150 mL of solution of SMX, ANT or TRZ (10 mg/L) and 2.4–2.7 g of avocado seed biochar (AS-Bc), 1.5–1.8 g of scraped avocado seed biochar (SAS-Bc), and 0.5 g of avocado exocarp biochar (AEx-Bc), 0.5 g of avocado endocarp biochar (Aen-Bc), 0.5 g of olive pit biochar (OP-Bc), and 0.5 g of cherry stone biochar (CS-Bc), stirred at 150 rpm for 120 or 150 min at room temperature (20 °C) and natural pH. In experiments involving granulated biochar (AEn-BC and AEx-BC), the material was mechanically crushed and sieved to achieve a particle size between 1 and 3 mm. After the assay time, an aliquot (1 mL) was collected and centrifuged (14,500 rpm for 10 min at 4 °C) using a Heraeus Fresco 21 Microcentrifuge (Thermo Fisher Scientific, Waltham, MA, USA). The filtered supernatant was analyzed by HPLC to determine the concentration of SMX, ANT and TRZ. All tests were accomplished in triplicate, and a blank test was run for each test performed.

#### 2.5.2. Adsorption Kinetics

The kinetic biochar adsorption studies were performed in 200 mL flasks, with 150 mL of solution of SMX, ANT or TRZ (10 mg/L) and 0.5 g of AEx-Bc (granulated with a particle size between 1 and 3 mm), and 0.5 g of olive pit biochar (OP-Bc), stirred at 150 rpm for 150 min at room temperature (20 °C) and natural pH. After the assay time, an aliquot (1 mL) was collected and centrifuged (14,500 rpm for 10 min at 4 °C) using a Heraeus Fresco 21 Microcentrifuge (Thermo Scientific). The filtered supernatant was analyzed by HPLC to determine the concentration of SMX, ANT and TRZ. All tests were accomplished in triplicate, and a blank test was run for each test performed.

Two commonly used kinetic models were selected—Pseudo-first and Pseudo-second order (Equations (1) and (2), respectively)—to fit the experimental values.

(1) $$d_{q}/d_{t}=k_{1}\cdot (q_{e}{-q}_{t})$$

(2) $$d_{q}/d_{t}=k_{2}\cdot (q_{e}-{q_{t})}^{2}$$

where d_q_/d_t_ is the adsorption rate; k_1_ is the pseudo-first order rate constant (1/min); q_e_ is the adsorption capacity at equilibrium (mg/g); q_t_ is the amount of solute adsorbed at time t (mg/g); k_2_ is the pseudo-second order rate constant (g/mg·min).

#### 2.5.3. Adsorption Equilibrium Studies

The equilibrium biochar adsorption studies were performed in 200 mL flasks, with 150 mL of solution of SMX, ANT or TRZ (10 mg/L) and between 0.25 and 1.5 g of AEx-Bc (granulated with a particle size between 1 and 3 mm), and between 0.25 and 1.5 g of OP-Bc, stirred at 150 rpm for 150 min at room temperature (20 °C) and natural pH. After the assay time, an aliquot (1 mL) was collected and centrifuged (14,500 rpm for 10 min at 4 °C) using a Heraeus Fresco 21 Microcentrifuge (Thermo Scientific). All tests were accomplished in triplicate, and a blank test was run for each test performed.

Two commonly used isotherm models were selected—Langmuir and Freundlich (Equations (3) and (4), respectively)—to fit the experimental values.

(3) $$q=q_{max}\cdot K_{L}\cdot C/({1+K}_{L}\cdot C)$$

(4) q=KF·C1n

where q_max_ is the maximum monolayer adsorption capacity (mg/g), K_L_ is the Langmuir equilibrium constant related to the affinity of the binding sites (L/mg), C is the equilibrium concentration of adsorbate in the solution (mg/L); K_F_ is the Freundlich adsorption capacity constant (mg^1−n^L^n^/g), *n* is the adsorption intensity or surface heterogeneity parameter (dimensionless).

### 2.6. Real Wastewater Matrix

Real wastewater samples were supplied by a municipal wastewater treatment plant situated in Tui (Augas do Louro, South of Galicia, Spain). The principal physicochemical parameters of the effluent are presented in Table 2.

## 3. Results

### 3.1. Screening Adsorption Experiments

Adsorption experiments were carried out using an initial pollutant concentration of 10 mg/L. This concentration was selected to enable a thorough evaluation of the adsorption behavior of the developed materials toward the selected model contaminants. Although it is not representative of the concentrations typically detected in aquatic environments, these pharmaceuticals have been reported at environmentally relevant levels. For example, TRZ has been detected at concentrations of up to 325 ng/L in Brazil [8], while SMX has been reported at concentrations reaching 600 ng/L in Portugal [24]. In the case of ANT, lower concentrations have been reported (approximately 2 ng/L); however, its detection in drinking water [25] highlights its environmental relevance and the need for its effective removal.

**Figure 2:**
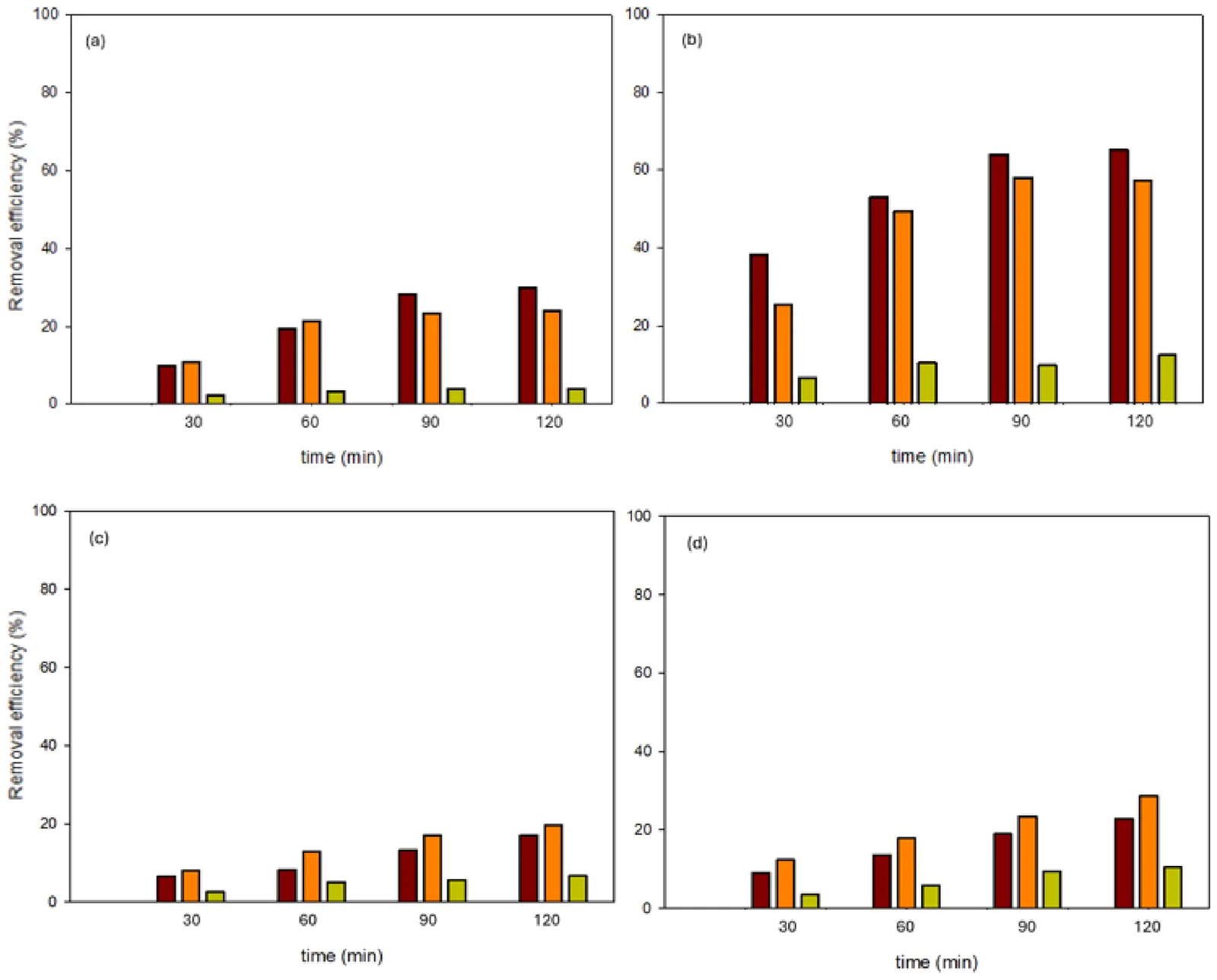
Adsorption, expressed in terms of removal percentage, of the different contaminants (SMX, represented by maroon bars; ANT, represented by orange bars; and TRZ, represented by mustard-colored bars) onto AS-Bc (**a**); SAS-Bc (**b**); AEn-Bc (**c**); and granular AEn-Bc (**d**).

The first adsorption tests were carried out using AS-Bc. Using this material, adsorption experiments were conducted separately for the three model contaminants: SMX, TRZ, and ANT. The selection of these model pharmaceuticals aimed to represent contaminants in their three ionic forms: anionic, cationic, and neutral. Specifically, at the working pH of the solutions (natural pH around 6), SMX is predominantly negatively charged [26], TRZ presents a positively charged species [11], and ANT remains neutral [26] (pK_a_ data are reported in Table 1). As mentioned above, each pharmaceutical was adsorbed individually onto AS-Bc, and the corresponding results are shown in Figure 2a.

As shown in Figure 2a, the adsorption results, expressed as removal percentage after a contact time of 120 min, exhibit significant differences among the studied contaminants. The highest removal was obtained for SMX, reaching approximately 30%, followed by ANT with 24%, whereas TRZ showed a very limited removal of around 4%.

These differences reflect a marked variation in the affinity of the different adsorbates toward the AS-Bc, which can be mainly attributed to their distinct physicochemical properties, such as ionization state, polarity, and ability to interact with the functional groups present on the adsorbent surface [27]. In particular, the low removal observed for TRZ suggests weak interactions with the biochar under the applied operating conditions [28]. Overall, these results evidence the limited adsorption capacity of the proposed material for certain compounds, highlighting the need to evaluate other avocado waste fractions.

In this context, and following the same objective as the previous experiment, other avocado waste fractions were recovered (Figure 1) for the removal of the pollutants, specifically the avocado seed, this time using the inner part (used whole), the skin covering the seed, referred to as the endocarp (used both whole and in granular form), and the outer peel, known as the exocarp (used only in granular form).

The next material evaluated for adsorption was the biochar obtained from the inner fraction of the avocado seed, after mechanically removing the outer layer of the seed biochar, which had been used in the initial adsorption experiment (Figure 2b). This approach was adopted based on the observation that the inner fraction exhibited a higher density of apparent cavities and could therefore present increased porosity and affinity for adsorption-based treatment [29].

Accordingly, the SAS-Bc was employed in the adsorption experiments of the three contaminants individually, as shown in Figure 2b. The adsorption results, expressed as removal percentage after the same contact time used in the initial experiment (120 min), follow the same relative trend among the contaminants, but with markedly higher removal values. The highest removal was again obtained for SMX, reaching approximately 65%, followed by ANT with 57%, while TRZ exhibited a more limited removal, on the order of 12%.

These results demonstrate a significant improvement in adsorption performance compared to the initial experiments, indicating an enhanced affinity between the contaminants and the modified adsorbent. In comparative terms, the achieved removal percentages are two to three times higher than those previously obtained, suggesting that the removal of the outer layer of the seed biochar promotes the exposure of active sites and enhances surface–adsorbate interactions, thereby increasing the overall efficiency of the adsorption process [30,31].

In the same framework, pyrolyzed residues from other avocado-derived fractions were systematically investigated to achieve higher adsorption efficiencies. The next fraction assessed was the layer covering the avocado seed, referred to as the endocarp. This material could be pyrolyzed while preserving its original structure and it has been tested in adsorption (Figure 2c); additionally, an AEn-Bc was also prepared in granular form and evaluated in adsorption (Figure 2d). This approach enabled the evaluation of the effect of particle morphology on adsorption performance [32], an aspect that would be considerably more challenging to investigate in the case of the seed due to its higher mechanical hardness.

As illustrated in Figure 2c, the use of AEn-Bc did not result in an improvement in removal efficiency compared to the previously tested materials. A slight enhancement was observed when the granular form (Figure 2d) was employed (in comparison with the whole biochar); however, the overall adsorption performance remained limited. It should be noted that, to ensure a rigorous comparison, the same adsorbent masses were used as in the experiments conducted with the SAS-Bc. As can be observed, the removal efficiencies achieved using the whole AEn-Bc were 17% for SMX, 20% for ANT, and 7% for TRZ. In contrast, adsorption using the granular AEn-Bc resulted in higher removal values of 23%, 29%, and 11% for SMX, ANT, and TRZ, respectively, corresponding to an approximate 1.5-fold increase compared to the intact endocarp.

**Figure 3:**
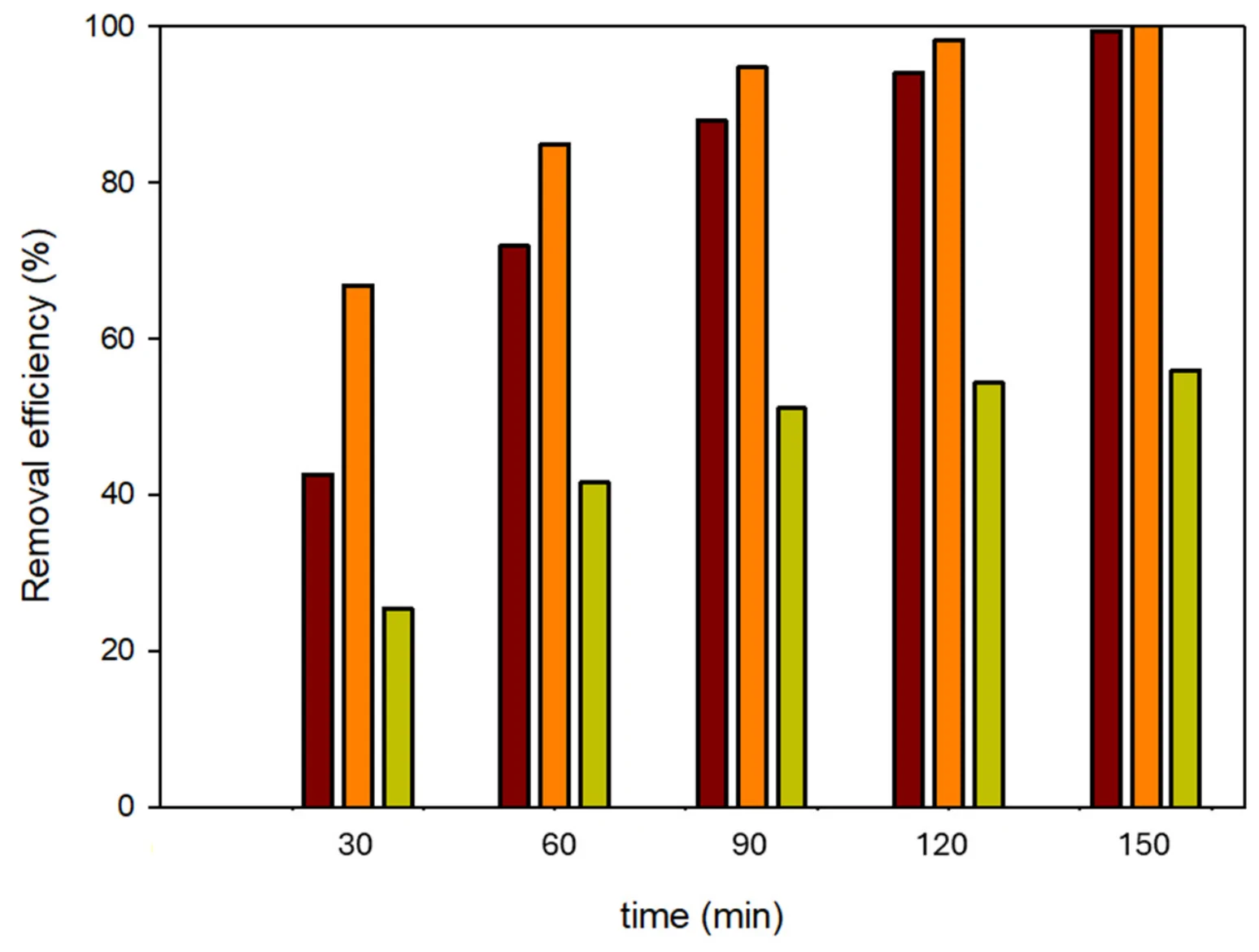
Adsorption, expressed in terms of removal percentage, of the different contaminants (SMX: maroon bars; ANT: orange bars; and TRZ: mustard-colored bars) onto AEx-Bc.

Finally, and following the approach of employing granular biochars, which are considerably easier to manufacture, adsorption experiments were conducted using biochar derived from the AEx (i.e., the outermost peel of the fruit; the previously studied peel corresponded to the layer covering the seed: endocarp, as shown in Figure 2). In this case, the contact time was again fixed at 120 min, consistent with all previous experiments, and the same adsorbent dosage was applied. As illustrated in Figure 3, a substantial enhancement in the adsorption of all three pharmaceuticals was observed. Notably, removal efficiencies approached 100% for two of the three contaminants.

SMX exhibited a marked increase in adsorption, reaching a removal efficiency of 94%. Similarly, ANT removal increased to 98%, while TRZ removal reached approximately 56%. Considering the results obtained in the previous experiments, AEx-Bc can be identified as the most effective adsorbent among all the avocado-derived fractions evaluated in this study. However, despite representing the highest TRZ removal achieved thus far, the elimination remained slightly above 50%, which is still considered insufficient from a remediation perspective when using adsorption-based treatments. This limited performance toward TRZ may be associated with weaker adsorbent–adsorbate interactions [33], differences in molecular structure and hydrophobicity [34], or a lower affinity of TRZ for the surface functional groups [35] present on AEx-Bc under the studied conditions.

**Figure 4:**
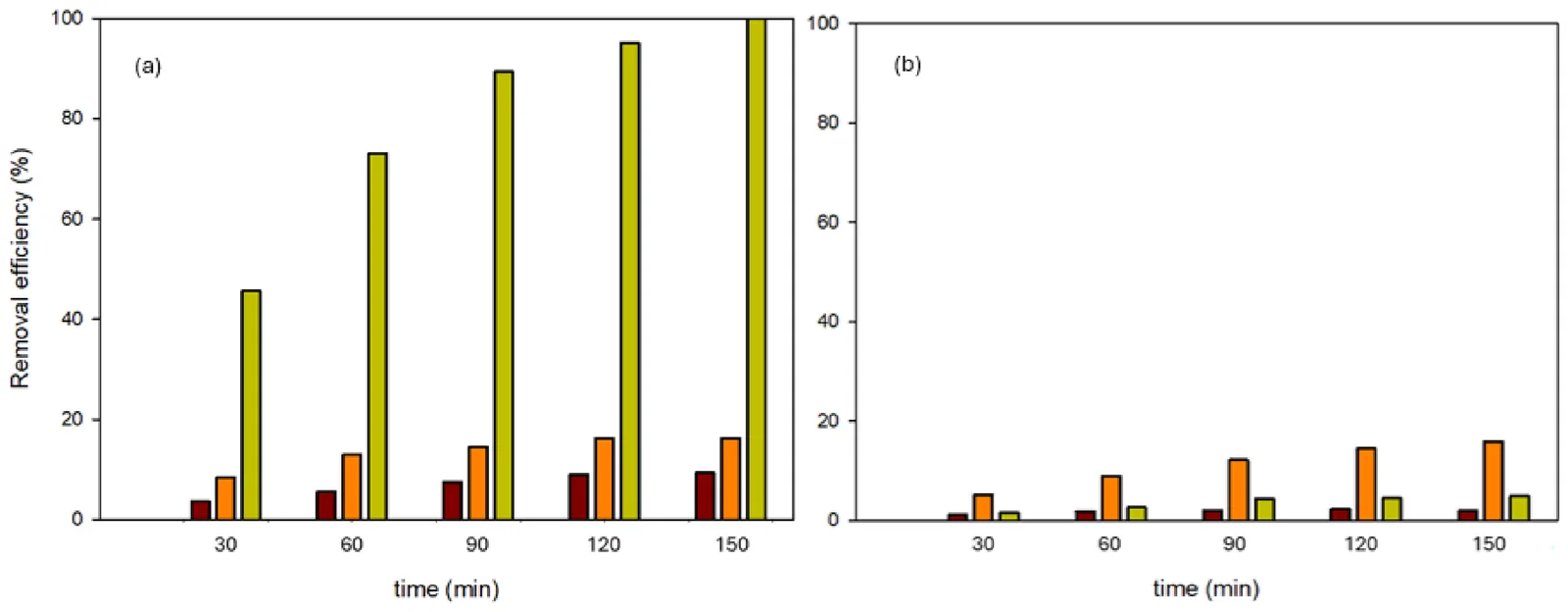
Adsorption, expressed in terms of removal percentage, of the different contaminants (SMX, represented by maroon bars; ANT, represented by orange bars; and TRZ, represented by mustard-colored bars) onto OP-Bc (**a**) and CS-Bc (**b**).

Therefore, in order to achieve TRZ removal efficiencies comparable to those obtained for SMX and ANT, biochars derived from other agri-food seed wastes capable of providing large quantities of residual material were investigated, specifically OP (Figure 4a) and CS (Figure 4b). Similar to the avocado residues, these materials constitute abundant and low-cost wastes, in the case of olive pits, 450,000 tons annually in Spain [36,37], aligning with the sustainability-driven approach of this study. Although the subsequent experiments were primarily focused on TRZ adsorption, additional tests were also conducted with SMX and ANT to assess the overall effectiveness of these biochars toward all three target contaminants considered in this work.

It can be observed that CS-Bc exhibited low adsorption performance for all the studied contaminants, with removal efficiencies as low as 2% for SMX, 16% for ANT, and 5% for TRZ. In contrast, OP-Bc showed a markedly different behavior, achieving complete removal of TRZ (≈100%) within the same contact time applied throughout this comparative study (120 min). However, adsorption of the other two contaminants onto OP-Bc remained limited, with removal values of 9% for SMX and 16% for ANT.

At this stage, the results clearly indicate a strong selectivity among the evaluated adsorbents: AEx-Bc demonstrated high affinity toward SMX and ANT, whereas OP-Bc exhibited outstanding performance for TRZ removal. This contrasting adsorption behavior highlights the relevance of adsorbent surface properties and adsorbate-specific interactions in governing removal efficiency [38]. Consequently, a detailed characterization of these two selected biochars (AEx-Bc and OP-Bc) was carried out in order to elucidate the underlying mechanisms responsible for the different affinities observed between the adsorbents and the target contaminants. The results of this characterization are presented and discussed in the following section.

### 3.2. Characterization

To gain insight into the markedly different removal efficiencies observed, the first step consisted of determining the point of zero charge (pH_pzc_: Table 3) of the different biochars: AEx-Bc and OP-Bc.

The pH_pzc_ determined for AEx-Bc (8.91) provides a plausible explanation for the adsorption results, since under the working pH conditions the biochar surface is predominantly positively charged. Consequently, SMX, which is mainly present in its anionic form, is expected to exhibit the highest adsorption affinity due to electrostatic attraction, followed by ANT, which remains neutral, and finally TRZ, whose adsorption is hindered by electrostatic repulsion as it is positively charged, similar to the biochar surface [39].

In contrast, OP-Bc and CS-Bc exhibit lower pH_pzc_ values than the avocado-derived materials. In the case of OP-Bc, the pH_pzc_ of 4.53 indicates that, at the solution pH (≈6), the surface of the pyrolyzed material is negatively charged. Under these conditions, SMX is also negatively charged, ANT remains neutral, and TRZ is positively charged. This charge distribution results in a strong electrostatic attraction toward TRZ, as evidenced in Figure 4 by the high removal efficiency achieved (≈95%), which is significantly higher than that observed for the other two contaminants, whose removals did not exceed 20%.

Once the general adsorption behavior had been rationalized based on the pH_pzc_ values of the different biochars and their interaction (or lack of interaction) with the ionic state of the contaminants, the next step involved the analysis of the BET surface area of the most relevant biochars. As can be observed from the data reported in Table 3, the BET surface areas of the produced biochars are relatively low, indicating the absence of highly developed porous structures [40]. It is common to find these types of BET area values in materials such as biochar [41]. In this case, based on these results, it is evident that physical surface area is not the dominant factor governing the adsorption process in the systems investigated [42]. Instead, other parameters must play a more relevant role in controlling adsorption performance.

**Figure 5:**
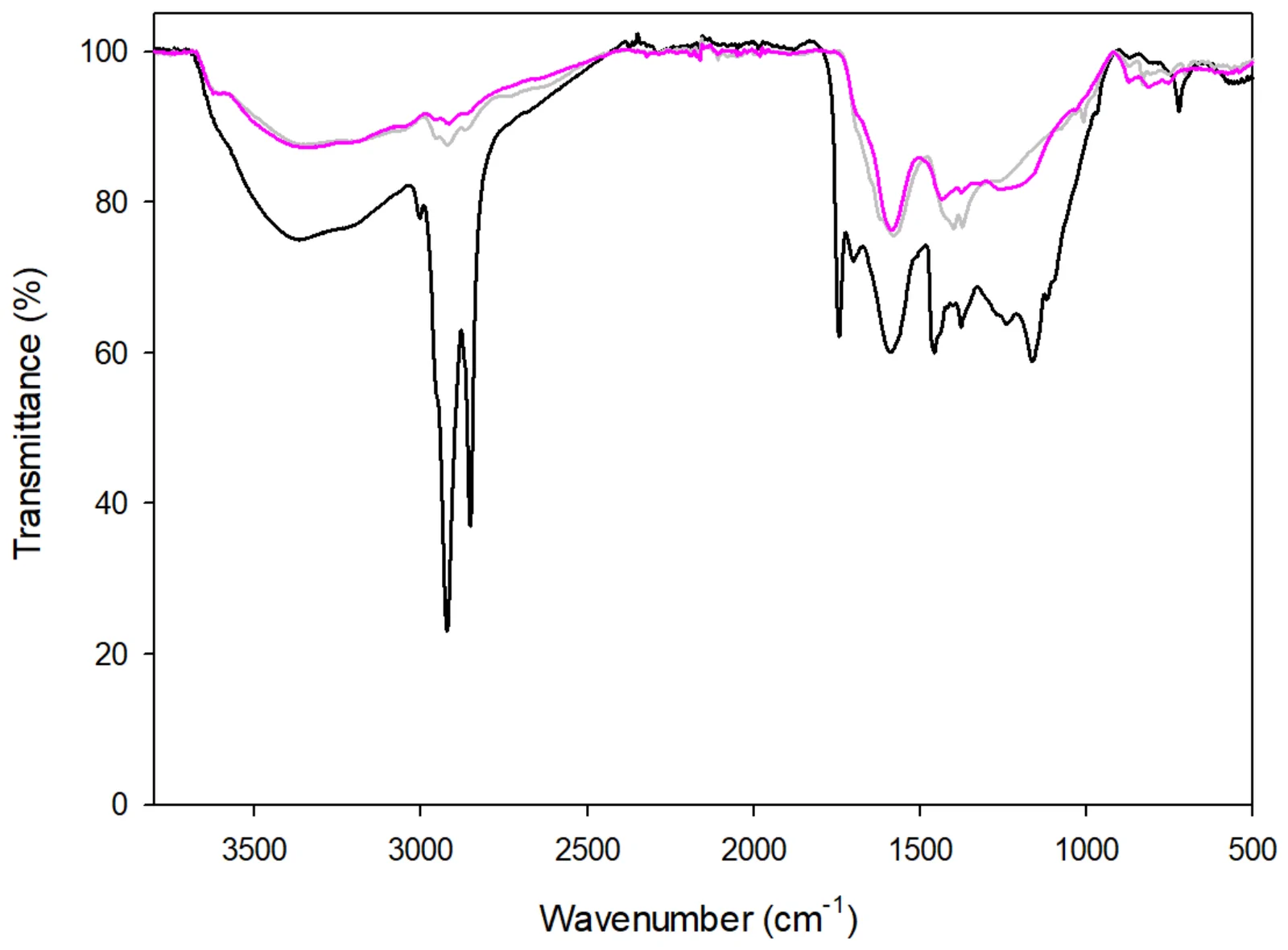
FTIR spectra of the different biochar samples: AEx-Bc (black line), SAS-Bc (gray line), and OP-Bc (pink line).

Although electrostatic attractions may partially explain the adsorption of SMX onto AEx-Bc and the strong affinity observed between OP-Bc and TRZ, the adsorption of ANT and TRZ onto avocado-derived biochars cannot be solely attributed to electrostatic interactions. To gain deeper insight into the mechanisms involved, Fourier-transform infrared (FTIR) analysis was performed on the two selected adsorbents (Figure 5). This characterization aimed to elucidate the interactions responsible for the nearly complete removal of ANT in its neutral form, as well as the contribution of surface functional groups to the adsorption process. A detailed characterization of AS-Bc, AEn-Bc and CS-Bc was not pursued due to their poor adsorption performance and limited relevance for further application.

The FTIR spectrum of SAS-Bc exhibits a broad O–H stretching band (~3600–3200 cm^−1^), indicating a significant abundance of hydroxyl groups capable of participating in hydrogen bonding and polar interactions [43]. Bands in the 1700–1600 cm^−1^ region confirm the presence of carbonyl functionalities and aromatic structures [44,45], which are known to participate in interactions with organic pollutants and metal species [46]. Additionally, signals between 1200 and 1000 cm^−1^ correspond to C–O vibrations, indicating a surface enriched in oxygen-containing functional groups [47]. Overall, SAS-Bc presents a functionalized chemical surface with numerous potential adsorption sites for polar contaminants in aqueous environments [48].

Among the evaluated materials, AEx-Bc displays the most intense functionalization. The O–H stretching band (~3600–3200 cm^−1^) is particularly pronounced, indicating a high density of hydroxyl groups [49]. In the aliphatic region (~2950–2850 cm^−1^), more distinct bands suggest the presence of residual aliphatic chains, which may contribute to hydrophobic interactions [50]. Carbonyl and aromatic bands in the 1700–1600 cm^−1^ region are also clearly visible, highlighting the potential of this material to interact with ionic or polar contaminants [51]. Furthermore, the intense signals observed between 1200 and 1000 cm^−1^ indicate a high concentration of C–O functionalities such as phenols, ethers and alcohols [52]. These features suggest that AEx-Bc possesses the highest density of oxygenated functional groups, which may enhance its adsorption capacity toward polar contaminants, heavy metals and dyes in aqueous systems [47].

The FTIR spectrum of OP-Bc shows lower intensity in the O–H stretching region (~3600–3200 cm^−1^), suggesting a lower density of hydroxyl groups compared to the other materials [53]. Similarly, the bands associated with carbonyl groups (1700–1600 cm^−1^) and C–O vibrations (1200–1000 cm^−1^) are less pronounced, indicating a surface with fewer oxygenated functionalities [47]. This spectral profile suggests a more carbonaceous surface with lower polarity, which may favor interactions with aromatic or less polar organic compounds rather than strongly polar species.

The adsorption results obtained for the emerging contaminants evaluated in this study show a clear relationship with the surface chemistry inferred from the FTIR spectra. In general, AEx-Bc and OP-Bc exhibited the best adsorption performance, consistent with their surface characteristics [54]. For SMX, the highest removal efficiency (≈98%) was obtained with AEx-Bc. This behavior can be attributed to electrostatic interactions between ionized SMX species and the abundant oxygen-containing groups identified in the FTIR spectrum, as well as hydrogen bonding and surface complexation mechanisms [55].

ANT adsorption also reached high values (≈95%) on AEx-Bc. Since electrostatic interactions are not expected to dominate under the experimental conditions, the process is likely governed by hydrogen bonding, dipole–dipole interactions and possible π–π interactions between the aromatic structures of the biochar matrix and the ANT molecule [56].

In contrast, TRZ exhibited the highest removal efficiency with OP-Bc, reaching complete adsorption (≈100%). This behavior suggests that electrostatic interactions between TRZ and the adsorbent surface play a dominant role under the experimental conditions [57]. Although OP-Bc contains fewer oxygenated groups than AEx-Bc, its surface chemistry appears to favour stronger interactions with TRZ. Conversely, AEx-Bc achieved approximately 60% removal of TRZ, likely governed by weaker mechanisms such as hydrogen bonding or hydrophobic interactions.

These results indicate that adsorption efficiency depends not only on the abundance of surface functional groups but also on the chemical nature of the contaminant and the dominant interaction mechanisms involved, including electrostatic attraction, hydrogen bonding, dipole–dipole interactions and π–π stacking. FTIR characterization therefore provides valuable insight into the differential adsorption behavior observed for each pharmaceutical compound across the evaluated agri-food waste-derived biochars.

While FTIR analysis provided valuable insight into the surface chemical composition and the nature of the functional groups responsible for the adsorption of the target pharmaceuticals, it does not fully account for the role of surface accessibility and structural features in the adsorption process. Therefore, to complement the chemical characterization and to better understand how surface morphology and porosity may influence adsorption performance, a morphological analysis of the selected biochars was carried out by SEM. This approach enables the correlation of adsorption behavior with structural attributes such as surface roughness, pore architecture, and the accessibility of active sites, providing a more comprehensive interpretation of the observed adsorption efficiencies.

**Figure 6:**
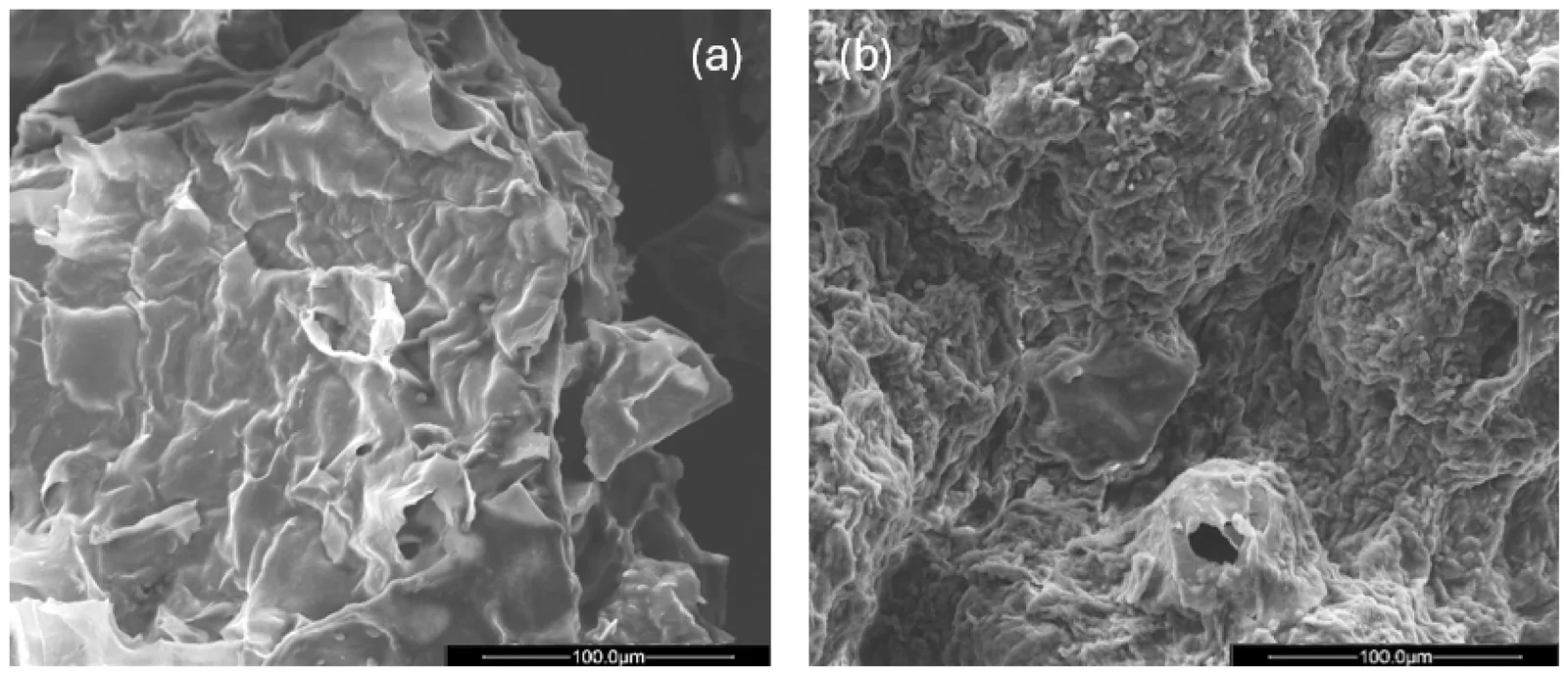
SEM images of AEx-Bc (**a**) and OP-Bc (**b**), at a scale of 100 μm.

The SEM micrograph of AEx-Bc (Figure 6a) reveals a morphology dominated by folded and collapsed laminar structures, indicative of a largely amorphous carbon matrix, which is characteristic of biochars derived from lignocellulosic residues [58]. This morphology is consistent with the irregular carbonaceous structure typically observed in lignocellulosic biochars. Although SEM does not provide direct information on the degree of aromatic ordering, the presence of carbon-rich laminar structures, together with the FTIR results, suggests that π–π interactions may contribute to the adsorption of aromatic pharmaceutical molecules. The presence of irregular cracks and cavities indicates the development of a porous structure that may facilitate mass transfer towards the internal adsorption sites, in which macropores and mesopores act as transport pathways toward internal active sites, while the contribution of microporosity is supported by the BET analysis rather than directly observable in the SEM images [59]. In addition, the relatively smooth contours observed in some laminar regions may indicate partial fusion or sintering during the pyrolysis process. This feature may be associated with a limited development of fine porosity, which is consistent with the BET results [60].

The SEM of OP-Bc (Figure 6b) reveals a highly heterogeneous and markedly rough surface, characterized by an irregular morphology with abundant cavities and collapsed laminar structures. Micrometer-sized pores, on the order of several tens of micrometers, are clearly visible, along with open cavities of irregular geometry, indicating the development of macroporosity associated with the thermal decomposition of lignocellulosic components during pyrolysis [61]. The observed morphology suggests that OP-Bc possesses a porous and heterogeneous surface that is favourable for adsorption applications in aqueous media, combining structural accessibility with an irregular external surface that can facilitate both physical adsorption and interactions between the adsorbate molecules and the surface functional groups.

### 3.3. Adsorption Kinetics

**Figure 7:**
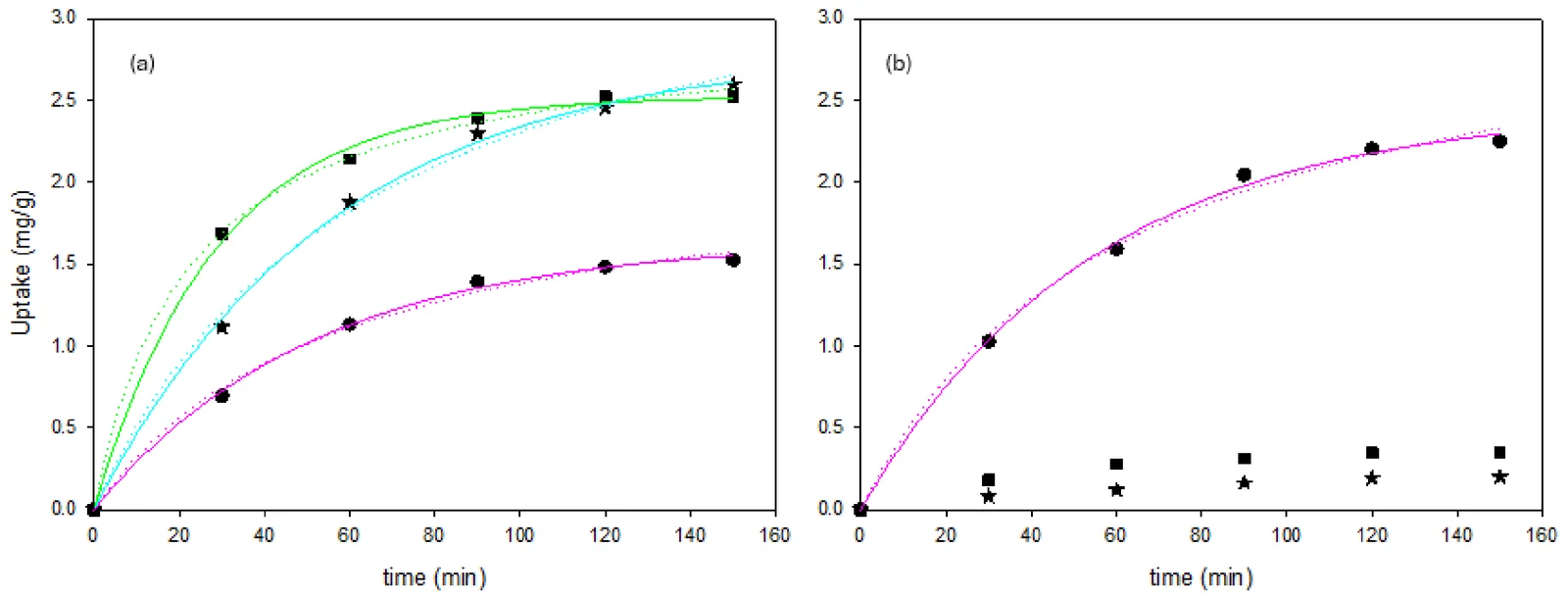
Adsorption kinetics of the different contaminants (SMX, represented by stars; ANT, represented by squares; and TRZ, represented by circles) onto AEx-Bc (**a**) and OP-Bc (**b**). Solid lines represent the fit to the pseudo-first order model, while dotted lines represent the fit to the pseudo-second order model.

Once the most effective biochar adsorbents for the target contaminants were identified, namely AEx-Bc for SMX and ANT, and OP-Bc for TRZ, a kinetic study was carried out for the three adsorption systems. To this end, the experiments were conducted over extended contact times beyond the previously fixed 120 min, to ensure that adsorption reached the total removal. Figure 7a presents the adsorption kinetics expressed in terms of uptake capacity for the three contaminants onto AEx-Bc. As expected, when the contact time was extended, complete removal of both contaminants (SMX and ANT) was achieved, with equilibrium reached at approximately 150 min. In contrast, TRZ adsorption did not reach equilibrium within the same time frame, although a clear deceleration in the adsorption rate was observed, as reflected by a marginal increase of only about 2% between 120 and 150 min. This behavior indicates a substantially lower affinity of TRZ for AEx-Bc compared to SMX and ANT.

To further elucidate the adsorption mechanism, the experimental kinetic data were analyzed using the pseudo-first order and pseudo-second order kinetic models. Table 4 summarizes the kinetic parameters obtained from the pseudo-first order and pseudo-second-order models for the adsorption of SMX, ANT, and TRZ onto AEx-Bc, as well as TRZ onto OP-Bc. A clear trend can be observed when comparing the calculated equilibrium adsorption capacities (q_e_) with the experimental values (q_e_ experimental). For all evaluated systems, the pseudo-first order model provides q_e_ values in close agreement with the experimental data, whereas the pseudo-second order model systematically overestimates the equilibrium capacities.

For instance, in the case of SMX adsorption onto AEx-Bc, the experimental q_e_ value (2.598 mg g^−1^) is well reproduced by the pseudo-first order model (2.803 mg g^−1^), whereas the pseudo-second order model overestimates the value (3.801 mg g^−1^). A similar trend is observed for ANT and TRZ adsorption onto AEx-Bc and for TRZ onto OP-Bc, indicating that the pseudo-second order model does not accurately represent the adsorption behavior under the studied conditions.

Although both models show high correlation coefficients (R^2^ ≥ 0.99), error analysis further confirms the superiority of the pseudo-first order model. Except for the RMSE value obtained for TRZ adsorption onto AEx-Bc, all other RMSE and χ^2^ values are consistently obtained for this model, indicating better agreement between experimental and predicted data.

The better fit of the pseudo-first order model indicates that this model provides a more appropriate mathematical description of the adsorption kinetics under the conditions investigated. However, the predominance of this model should not be interpreted, by itself, as direct evidence of physisorption, chemisorption, or a specific molecular interaction mechanism, since kinetic models primarily describe the temporal evolution of adsorption rather than directly identifying the molecular nature of the adsorbate–adsorbent interactions [62,63].

The observed kinetic behavior may instead be interpreted together with the surface characterization and the physicochemical properties of both the biochars and the pharmaceuticals. In this regard, the FTIR results revealed the presence of oxygen-containing functional groups (–OH, C=O and C–O), which may contribute to adsorption through hydrogen bonding, electrostatic interactions, and dipole–dipole interactions with the target contaminants [64]. These interactions, together with the heterogeneous surface structure observed by SEM, may contribute to the rapid initial uptake observed in the kinetic experiments, despite their relatively low BET surface areas [65].

Furthermore, the relatively high k_1_ values obtained (0.018–0.035 min^−1^) indicate favorable adsorption rates, with equilibrium reached within approximately 150 min for the most efficiently adsorbed compounds. Differences in adsorption performance among the pharmaceuticals therefore appear to be related mainly to variations in surface affinity rather than kinetic limitations.

**Figure 8:**
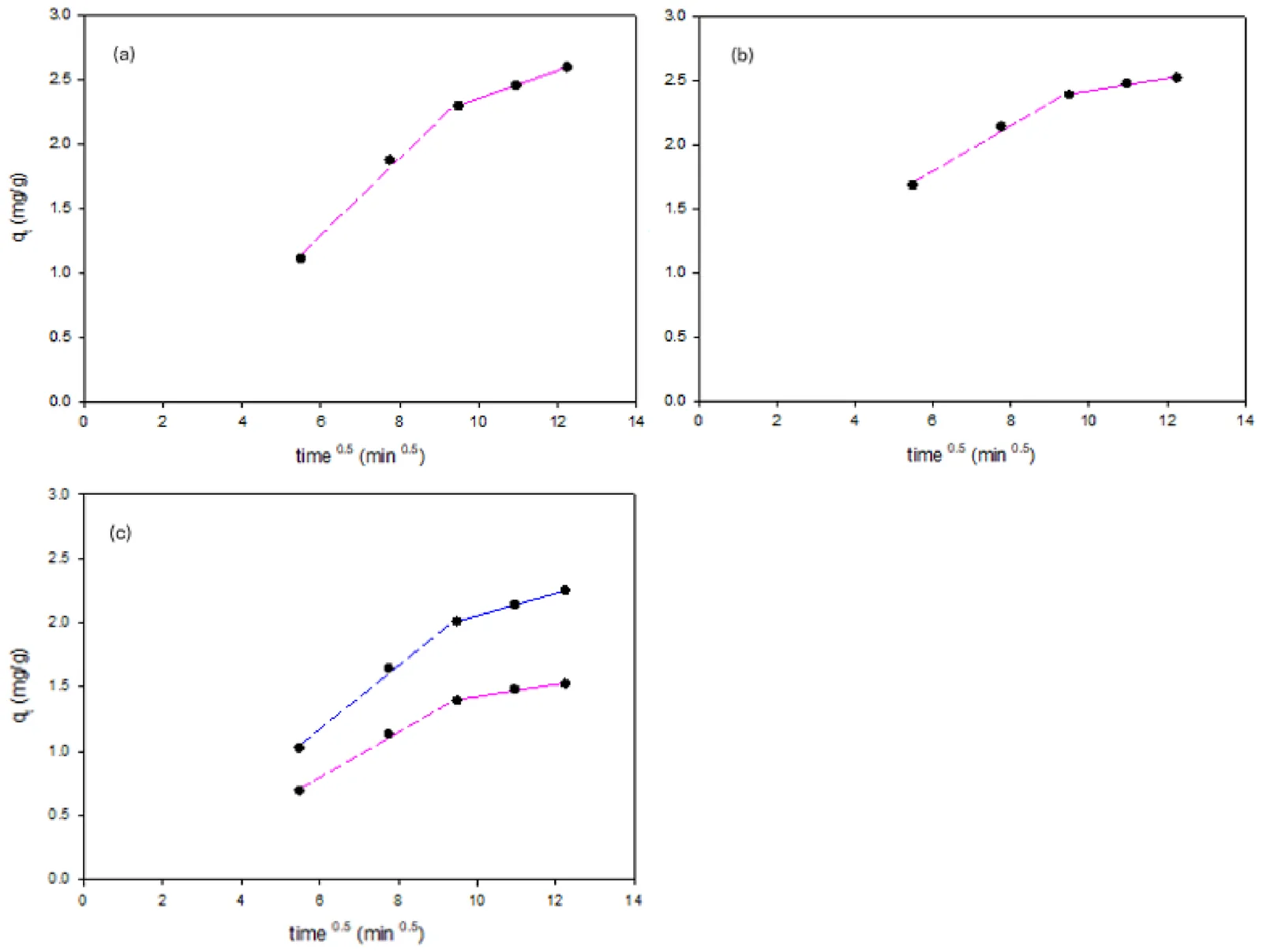
Intraparticle diffusion model fittings for adsorption onto AEx-Bc (pink line): SMX (**a**), ANT (**b**), and TRZ (**c**); adsorption of TRZ onto OP-Bc (blue line: (**c**)). The dotted line represents the first region and the solid line the second region of adsorption.

To further investigate the adsorption mechanism, the experimental data were also evaluated using the intraparticle diffusion model (Figure 8). This model was applied to SMX (Figure 8a), ANT (Figure 8b) and TRZ adsorption onto AEx-Bc (Figure 8c, pink lines) and OP-Bc (Figure 8c, blue lines). In all cases, plots of q_t_ versus t^1/2^ showed a multilinear behavior, indicating that adsorption proceeds through several kinetic stages rather than a single diffusion-controlled mechanism.

The first linear region, observed at short contact times (typically within the first 60 min), corresponds to rapid adsorption dominated by external mass transfer and surface interactions. During this stage, adsorption mainly occurs on the external surface of the biochars and is driven by electrostatic attraction in the case of SMX and by hydrogen bonding, dipole–dipole interactions and π–π interactions for neutral compounds such as ANT. This rapid uptake is consistent with the high density of oxygen-containing functional groups identified by FTIR.

The second linear region, appearing at longer contact times, shows a lower slope and corresponds to a slower approach to equilibrium, where intraparticle diffusion contributes to the overall adsorption process. However, the lines do not pass through the origin, as indicated by the non-zero intercepts, demonstrating that intraparticle diffusion is not the sole rate-limiting step and that boundary layer diffusion also plays an important role.

Clear differences were observed in TRZ adsorption between the two biochars. While AEx-Bc exhibited moderate adsorption due to partial electrostatic repulsion, OP-Bc achieved almost complete removal of TRZ, characterized by a pronounced initial uptake and rapid equilibrium. This behavior suggests a stronger affinity between TRZ and the OP-Bc surface, likely associated with differences in surface chemistry rather than textural properties. These results agree with the excellent fit obtained with the pseudo-first order model and further support that adsorption is controlled primarily by surface chemistry rather than internal diffusion. Based on these findings, equilibrium adsorption experiments were further analyzed using adsorption isotherm models to better characterize the interaction strength and adsorption behavior of the model contaminants.

### 3.4. Adsorption Isotherms

The equilibrium adsorption behavior of SMX, ANT, and TRZ was investigated using Langmuir and Freundlich isotherm models in order to elucidate the adsorption mechanisms governing the interaction between the target contaminants and the selected biochars. The experimental data were fitted to both models for the two selected biochar: AEx-Bc with the contaminants SMX, ANT, and TRZ; and OP-Bc with the contaminant TRZ. Table 5 summarizes the isotherm parameters obtained from the Langmuir and Freundlich models for the adsorption of SMX, ANT, and TRZ onto AEx-Bc, and TRZ onto OP-Bc. The analysis reveals significant differences in adsorption behavior depending on the contaminant–adsorbent system.

For SMX and ANT adsorption onto AEx-Bc, the Langmuir model provides an excellent fit to the experimental data, with high correlation coefficients (R^2^ = 0.994 and 0.999, respectively). The maximum adsorption capacities predicted by the model (q_max_ = 5.834 mg/g for SMX and 5.682 mg/g for ANT) are consistent with the experimental equilibrium values (4.191 and 5.644 mg/g, respectively), particularly in the case of ANT, where the agreement is nearly exact. The relatively high Langmuir constants (K_L_ = 0.955 L/mg for SMX and 1.469 L/mg for ANT) indicate strong affinity between the adsorbates and the active sites of the AEx-Bc. These results support a monolayer adsorption mechanism occurring on energetically homogeneous surface sites, consistent with the rich surface functionalization previously identified by FTIR analysis [66].

The Freundlich model also provides a good fit for these two systems (R^2^ ≥ 0.983), with n values greater than 1 (n = 2.099 for SMX and 2.350 for ANT), confirming favorable adsorption conditions. However, the slightly lower R^2^ values compared to the Langmuir model suggest that adsorption is better described by a finite number of well-defined active sites rather than by multilayer adsorption on a highly heterogeneous surface.

In contrast, TRZ adsorption onto AEx-Bc shows a markedly different behavior. Although the Langmuir model yields a reasonable correlation coefficient (R^2^ = 0.984), the negative q_max_ (−1.379 mg/g) and K_L_ (−0.114 L/mg) values lack physical meaning, indicating that the Langmuir assumptions are not suitable for describing this system. These unrealistic parameters suggest that TRZ adsorption cannot be adequately described as a homogeneous monolayer process occurring on energetically equivalent adsorption sites. Similarly, the Freundlich model produces a low n value (n = 0.440), which suggests unfavorable adsorption conditions and weak surface affinity. These findings are consistent with the lower removal efficiencies previously observed for TRZ onto AEx-Bc and confirm that adsorption does not follow an ideal monolayer adsorption mechanism.

For TRZ adsorption onto OP-Bc, the Langmuir model again provides an excellent fit (R^2^ = 0.997), with a high predicted maximum adsorption capacity (q_max_ = 10.756 mg/g), substantially exceeding the experimental equilibrium value (4.018 mg/g), indicating a strong theoretical adsorption potential. The positive KL value (0.257 L/mg) reflects favorable adsorbent–adsorbate interactions. The Freundlich model also shows a very good fit (R^2^ = 0.995) with n = 1.317, confirming favorable adsorption behavior. The good agreement between both models suggests that TRZ adsorption onto olive pit biochar may involve strong surface interactions combined with a certain degree of surface heterogeneity.

In general terms, the isotherm analysis confirms that adsorption performance strongly depends on the specific contaminant–biochar interaction. SMX and ANT adsorption onto AEx-Bc follows predominantly Langmuir-type behavior, indicating monolayer adsorption on well-defined active sites. In contrast, TRZ adsorption onto AEx-Bc deviates from classical isothermal assumptions, reflecting weak affinity and non-ideal adsorption behavior. Conversely, TRZ adsorption onto OP-Bc exhibits favorable and well-defined adsorption characteristics, consistent with the strong electrostatic interactions previously inferred from pH_pzc_ analysis. These results reinforce the conclusion that adsorption efficiency is governed primarily by the chemical compatibility between the contaminant and the surface functional groups of the biochar rather than solely by general surface area or textural properties. These results are in agreement with other published studies, such as the work by Li et al. [67] on the adsorption of SMX using biochar, as well as the conclusions reported by Qiu et al. [68] in their review on biochar as an adsorbent for organic contaminants in wastewater.

### 3.5. Validation in Real Wastewater Matrix

**Figure 9:**
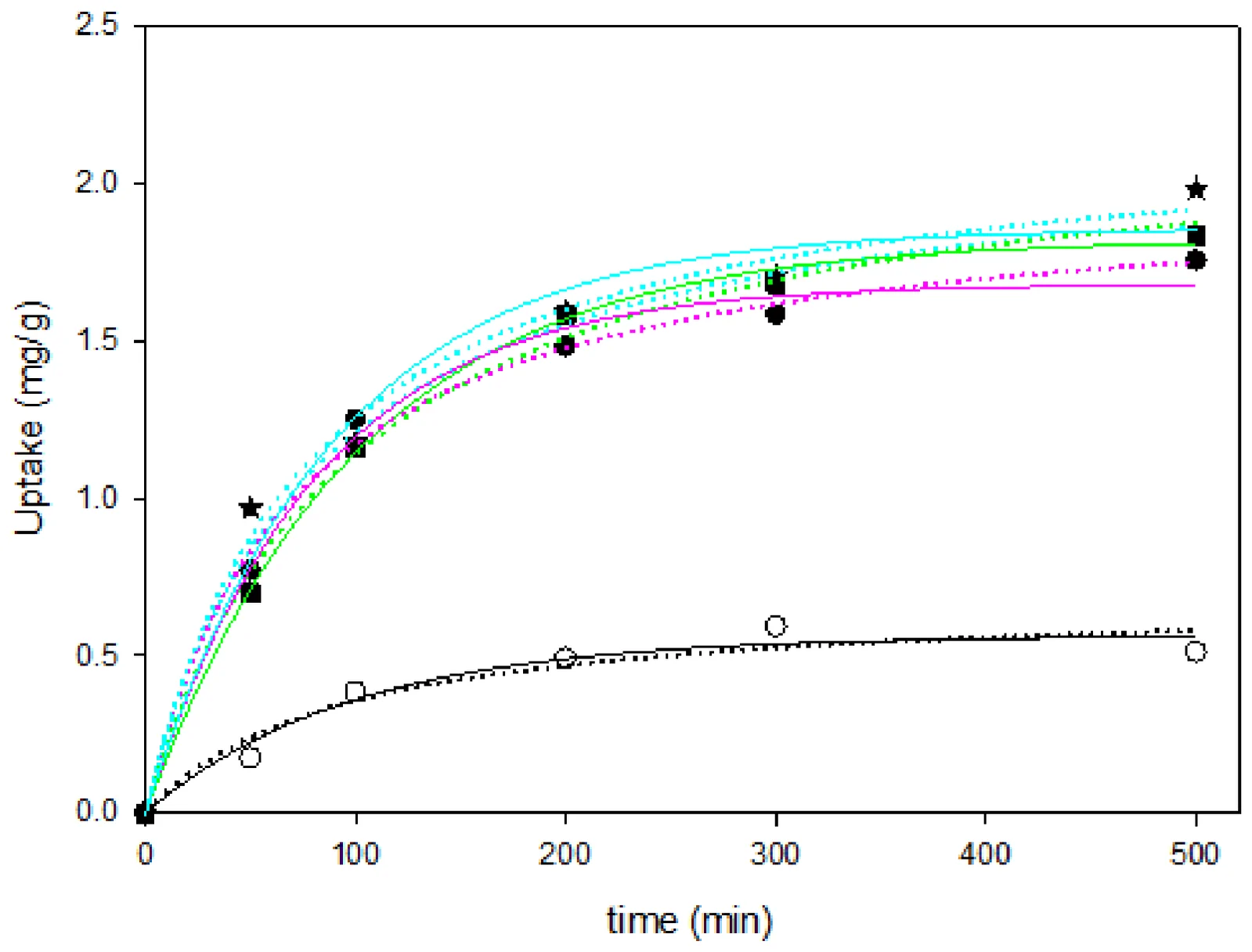
Adsorption kinetics of the different contaminants (SMX, represented by stars; ANT, represented by squares; and TRZ, represented by empty circles) onto AEx-Bc; and TRZ (represented by bold circles) onto OP-Bc. Solid lines represent the fit to the pseudo-first order model, while dotted lines represent the fit to the pseudo-second order model.

The adsorption performance of the selected biochars was finally evaluated using real wastewater collected in the inlet of a municipal WWTP (Augas do Louro, South of Galicia, Spain: characteristics in Section 2.6) in order to assess their effectiveness under realistic and complex conditions. As shown in Figure 9, SMX and ANT were treated using AEx-Bc, whereas TRZ was removed using OP-Bc, in accordance with the optimal adsorbent–adsorbate combinations identified throughout this study. The experimental equilibrium capacities (q_e_,exp) decreased notably, particularly for TRZ onto AEx-Bc (0.511 mg/g), confirming that competitive adsorption phenomena significantly affect low-affinity systems. In contrast, SMX and ANT onto AEx-Bc and TRZ onto OP-Bc maintained relatively stable adsorption performance despite the complex matrix, indicating a stronger intrinsic affinity toward these adsorbents.

Table 6 presents the kinetic parameters obtained for SMX, ANT, and TRZ adsorption onto AEx-Bc and OP-Bc using real wastewater from a WWTP. Compared to the experiments performed in synthetic solutions, a general decrease in equilibrium adsorption capacities is observed for all systems, which can be attributed to matrix effects inherent to real wastewater, including competition with dissolved organic matter (DOM), inorganic ions, and other coexisting micropollutants [69].

Regarding model fitting, both kinetic models provided reasonable correlation coefficients; however, the pseudo-first-order model generally yielded better agreement between calculated and experimental q_e_ values, especially for SMX and ANT onto AEx-Bc and TRZ onto OP-Bc. For example, in the case of ANT onto AEx-Bc, the calculated q_e_ (1.824 mg/g) closely matches the experimental value (1.838 mg/g), with an excellent R^2^ of 0.998 and very low RMSE and χ^2^ values. This indicates that adsorption under real wastewater conditions is still primarily governed by rapid surface interactions rather than by chemisorption-controlled mechanisms [70].

Although the pseudo-second order model shows slightly higher R^2^ values in some cases (e.g., SMX onto AEx-Bc), it consistently overestimates the equilibrium capacities and presents higher error values (RMSE and χ^2^), particularly for SMX and TRZ. This suggests that chemisorption is not the dominant rate-controlling mechanism, even in the presence of competing species.

The kinetic constants (k_1_ ≈ 0.010–0.012 min^−1^) remain within the same order of magnitude as those obtained in ultrapure water, indicating that the adsorption rate is not drastically hindered by the wastewater matrix. Therefore, the reduction in performance observed in real wastewater appears to be primarily thermodynamic (competition for active sites) rather than kinetic (diffusion limitation).

Notably, TRZ adsorption onto OP-Bc continues to exhibit strong kinetic performance (R^2^ = 0.991 for pseudo-first order), confirming that electrostatic attraction remains an effective driving force even under realistic environmental conditions. This highlights the robustness of OP-Bc as a selective adsorbent for TRZ in complex aqueous matrices.

The kinetic analysis in real wastewater confirms that surface chemistry remains the dominant factor controlling adsorption, but matrix effects significantly influence equilibrium capacity due to competitive adsorption phenomena. These results demonstrate the importance of validating adsorption performance under realistic conditions, as systems that perform well in synthetic solutions may exhibit reduced efficiency in real wastewater environments.

A direct comparison between the adsorption results obtained in ultrapure water and real WWTP influent highlights the significant influence of matrix complexity on pollutant removal. The real wastewater used in this study corresponded to untreated influent, characterized by high COD and BOD_5_ values, as well as the presence of dissolved organic matter, suspended solids, inorganic ions, and other competing compounds. In addition, experiments were performed at relatively high contaminant concentrations (≈10 ppm), representing a severely contaminated scenario [8,24]. Under these conditions, adsorption capacities decreased compared to synthetic solutions, mainly due to competition for the available active sites. Nevertheless, effective removal was still achieved, particularly for TRZ adsorption onto OP-Bc, demonstrating the robustness of the material even in highly challenging matrices.

Despite the reduction in adsorption performance, the overall adsorption behavior remained similar to that observed in ultrapure water, indicating that the fundamental removal mechanism was preserved. These findings suggest that the biochars maintained their active surface functionality even in the presence of high organic loads and complex wastewater constituents [71]. Therefore, the proposed materials show considerable potential for the treatment of highly contaminated wastewaters and industrial effluents, where adsorption could serve as an effective pre-treatment or polishing step within advanced water remediation strategies [72].

## 4. Conclusions

This study demonstrates that agri-food waste-derived biochars can act as effective and selective adsorbents for the removal of emerging pharmaceutical contaminants from water. AEx-Bc achieved near-complete removal of SMX and ANT, reaching 100% adsorption between 120 and 150 min, whereas OP-Bc enabled almost complete TRZ removal under the same contact time, highlighting the importance of matching adsorbent surface chemistry with contaminant properties.

Adsorption kinetics for all systems were best described by the pseudo-first order model, with calculated equilibrium capacities closely matching experimental values, indicating that adsorption is governed by fast and reversible surface interactions rather than by chemisorption or diffusion-controlled processes. Equilibrium isotherm analysis further confirmed that adsorption behavior is dominated by surface chemistry and adsorbent–adsorbate affinity rather than by textural properties such as BET surface area.

Surface characterization (FTIR and SEM) revealed that oxygen-containing functional groups and heterogeneous surface structures play a key role in promoting electrostatic interactions, hydrogen bonding, and π–π interactions. Importantly, the selected biochars maintained high removal efficiencies when applied to real municipal wastewater, demonstrating the robustness of the adsorption mechanisms in complex matrices. These results highlight the potential of avocado- and olive-derived biochars as sustainable, low-cost materials for tertiary wastewater treatment and support their application as tailored adsorbents for specific emerging contaminants.

Future work should focus on evaluating the long-term performance of these biochars through successive adsorption cycles to assess their stability and practical applicability under realistic operating conditions. In addition, a comprehensive physicochemical characterization before and after repeated use, including changes in surface functional groups, morphology, and textural properties, would provide valuable insight into the structural evolution of the materials and the mechanisms responsible for any changes in adsorption performance. Such studies will contribute to a better understanding of the durability and reusability of waste-derived biochars for sustainable water treatment applications.

## Figures and Tables

**Table 1:** Speciation and pK_a_ values of sulfamethoxazole (SMX), trazodone (TRZ) and antipyrine (ANT).

Sulfamethoxazole (SMX)	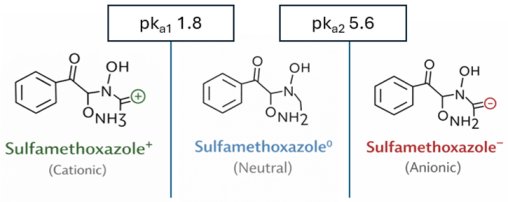
Trazodone (TRZ)	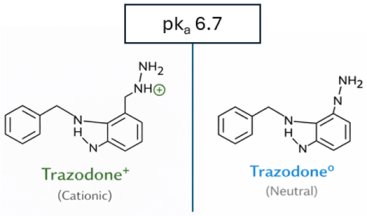
Antipyrine (ANT)	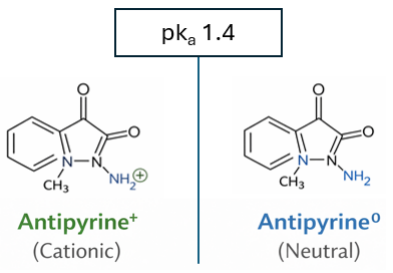

**Table 2:** Characteristics of untreated wastewater used in adsorption process (values in mg/L).

pH	Chemical Oxygen Demand (COD)	Biochemical Oxygen Demand (BOD_5_)	Suspended Solids (SS)	Total Nitrogen
7.10	312	117	92	17

**Table 3:** pH_pzc_ and BET surface area of AEx-Bc and OP-Bc.

Sample	pH_pzc_	BET Area (m^2^/g)
AEx-Bc	8.91	0.80
OP-Bc	4.54	1.13

**Table 4:** Adsorption model kinetics parameters evaluated in this study.

Model	Parameter	AEx-Bc: SMX	AEx-Bc: ANT	AEx-Bc: TRZ	OP-Bc: TRZ
Experimental	q_e_ (mg/g)	2.598	2.524	1.52	2.249
First-order kinetic model: Equation (1)	q_e_ (mg/g)	2.803	2.524	1.639	2.453
	k_1_ (1/min)	0.018	0.035	0.019	0.018
	R^2^	0.998	0.996	0.998	0.997
	RMSE	0.017	0.013	0.025	0.045
	χ^2^	0.004	0.004	0.003	0.002
Second-order kinetic model: Equation (2)	q_e_ (mg/g)	3.801	2.949	2.184	3.301
	k_2_ (g/mg min)	0.004	0.015	0.008	0.005
	R^2^	0.995	0.999	0.993	0.994
	RMSE	0.064	0.128	0.008	0.555
	χ^2^	0.013	0.433	0.318	2.721

**Table 5:** Adsorption model isotherms parameters evaluated in this study.

Model	Parameter	AEx-Bc: SMX	AEx-Bc: ANT	AEx-Bc: TRZ	OP-Bc: TRZ
Experimental	q_e_ (mg/g)	4.191	5.644	2.679	4.018
Langmuir: Equation (3)	q_max_ (mg/g)	5.834	5.682	−1.379	10.756
	K_L_ (L/mg)	0.955	1.469	−0.114	0.257
	R^2^	0.994	0.999	0.984	0.997
Freundlich: Equation (4)	K_F_ (mg^1−n^L^n^/g)	2.631	3.109	0.049	2.127
	n	2.099	2.350	0.440	1.317
	R^2^	0.983	0.999	0.983	0.995

**Table 6:** Adsorption model kinetics parameters with real wastewater matrix.

Model	Parameter	AEx-Bc: SMX	AEx-Bc: ANT	AEx-Bc: TRZ	OP-Bc: TRZ
Experimental	q_e_ (mg/g)	1.984	1.838	0.511	1.756
First-order kinetic model: Equation (1)	q_e_ (mg/g)	1.858	1.824	0.563	1.682
	k_1_ (1/min)	0.011	0.010	0.010	0.012
	R^2^	0.975	0.998	0.959	0.991
	RMSE	0.132	0.027	0.049	0.078
	χ^2^	0.099	0.002	0.023	0.011
Second-order kinetic model: Equation (2)	q_e_ (mg/g)	2.169	2.235	0.688	1.996
	k_2_ (g/mg min)	0.006	0.005	0.016	0.007
	R^2^	0.980	0.994	0.928	0.994
	RMSE	0.065	0.001	0.010	0.159
	χ^2^	0.047	0.161	0.094	1.127

## Data Availability

Data are contained within the article.
